# First isolation of *Campylobacter vicugnae* sp. nov. in humans suffering from gastroenteritis

**DOI:** 10.1128/spectrum.01523-24

**Published:** 2024-10-04

**Authors:** Quentin Jehanne, Lucie Bénéjat, Lamia Azzi Martin, Victoria Korolik, Astrid Ducournau, Johanna Aptel, Armelle Ménard, Marine Jauvain, Christophe Aguilera, Alice Doreille, Laurent Mesnard, Catherine Eckert, Philippe Lehours

**Affiliations:** 1 National Reference Centre for Campylobacters and Helicobacters, Bordeaux, France; 2 University of Bordeaux, Inserm, UMR 1312, Bordeaux Institute of Oncology (BRIC), Bordeaux, France; 3 Institute for Glycomics, Griffith University, Gold Coast, Australia; 4 Laboratoire d’Analyses Médicales Biosèvres, Bressuire, France; 5 Sorbonne Université, Inserm, U1135, Centre d’Immunologie et des Maladies Infectieuses, Paris, France; 6 AP-HP, Soins Intensifs Néphrologique et Rein Aigu, Hôpital Tenon, Paris, France; 7 Département de Bactériologie, AP-HP, Sorbonne-Université, Hôpital Saint-Antoine, Paris, France; Universidad Andres Bello, Santiago, Chile

**Keywords:** *Campylobacter *genus, emergent species, whole-genome sequencing, phylogeny

## Abstract

The present study describes the first isolation of a recently described *Campylobacter* species, *Campylobacter vicugnae*, in humans. The isolates were recovered by two independent French laboratories in 2020 and 2022 from a man and a woman suffering from gastroenteritis. Biochemical and growth characteristics, and electron microscopy for these two strains indicated that they belong to *Campylobacter* genus. *16S rDNA* and GyrA-based phylogeny, as well as average nucleotide identity and *in silico* DNA-DNA Hybridization analyses revealed that both strains belong to the *Campylobacter vicugnae* species. Both isolates possess a complete cytolethal distending toxin (CDT) locus with *cdtA*, *cdtB,* and *cdt*C, and features of CDT activity were demonstrated *in vitro* with Caco-2 intestinal epithelial cells. Our data suggest that these two isolates of *C. vicugnae* were associated with gastroenteritis in humans and induced major cytopathogenic effects *in vitro*. *C. vicugnae* is likely to be a novel human pathogen, with a source of foodborne infection that needs to be determined.

**IMPORTANCE:**

*Campylobacter* species that display toxicity features are a worldwide public health issue. In clinical contexts, it is crucial to identify which isolate could be an urgent threat to a patient. Actual and widely used laboratory methods such as mass spectrometry or PCR may be flawed in the field of species identification. In contrast, the present study shows that next-generation sequencing allows to precisely identify isolates to species level that may have been omitted otherwise. Moreover, it helps to identify emerging species before they become a threat to human health. Recovery of a new *Campylobacter* species in human sample, such as the new species “*Campylobacter vicugnae*,” is an important step for the identification of emerging pathogens posing threat to global health.

## INTRODUCTION

To date, the *Campylobacter* genus comprises 48 validated species and 13 subspecies (1), the most common species involved in human gastrointestinal disease being *Campylobacter jejuni* (2). Overall, campylobacteria are curved, spiral, or fusiform rods with size ranging from 1.5 to 10 µm long and 0.2 to 1.2 µm wide with polar flagella. The *Campylobacter* species are able to colonize alternative sites in the digestive tract of various hosts (mammals, birds, and reptiles). In the majority of cases, campylobacteriosis is the leading cause of human bacterial foodborne illness, linked to the consumption of contaminated chicken and ruminant meat (3–5). Potential sources of campylobacterial contamination are varied. In addition to contamination through meat consumption, unpasteurized milk, drinking water accidentally contaminated with animal droppings, and direct contact with the feces of farm animals or pets can cause campylobacteriosis. Some species, such as *C. fetus* subsp. *venerealis* (6), have never been described in humans, but others, such as *C. fetus* subsp. *fetus*, may be associated with opportunistic human infections, particularly in immunocompromised patients (7). Characterization of these cases is an important element in defining and understanding pathogenicity and epidemiology of various campylobacters.

Campylobacters share common morphological, cultural, and biochemical characteristics, notably their curved morphology, their mobility, and their need to grow in a preferential microaerobic atmosphere. Their size, their ability to grow at different temperatures, and their biochemical profiles can help the identification at species level. Nowadays, the advent of high-throughput sequencing has made it possible to access the genomes of these bacteria and better define their identity and phylogenetic relationship. Whole-genome analysis, including comparative genomics, is now an undisputed requirement for the characterization of any new species. Unfortunately, *16S rDNA* sequencing, a widely used tool for taxonomy, was shown to be unreliable for *Campylobacter* species identification as well as for other bacteria (8–11). In cases of suspected campylobacteriosis, the lack of appropriate identification is even more of concern, given that the vast majority of clinical laboratories are focusing on the identification of *C. jejuni* and *C. coli* only, thereby potentially compromising therapeutic decisions.

In recent years, modern genomics has made it possible to characterize new species of *Campylobacter* genus. In particular, the *Campylobacter lari* (12) group has been extensively studied, leading to the description of novel species, such as *C. armoricus* (13) and *C. ornithocola* (14). Miller et al. conducted, in 2017, comparative genomic analysis across the entire *Campylobacter fetus* group, especially *C. fetus* subsp. *fetus*, *C. fetus* subsp. *venerealis*, *C. hyointestinalis*, *C. iguanorium,* and *C. lanienae* (15). These findings included the identification of three putative novel *Campylobacter* species from particular clades belonging to *C. fetus* group termed *C. lanienae*. In 2023, isolates from clade 3 were identified in China in sheeps, and a novel species named “*Campylobacter ovis”* was proposed and awaited for validation (16). Miller et al. suggested, in 2024, three species names based on their previous data, which have been appropriately validated: *Campylobacter devanensis* sp. nov.*, Campylobacter porcelli* sp. nov.*,* and a new name for *C. ovis: Campylobacter vicugnae* sp. nov. (17). For clarity purposes, *C. ovis* isolates from China will here be referred as “*C. ovis-vicugnae*.”

The French National Reference Centre for Campylobacters and Helicobacters (NRCCH) receives *Campylobacter* isolates from private clinical laboratories and public hospitals. These isolates are sent for epidemiological tracing, and some are sent by investigators who failed to identify the bacteria at species level. All isolates are then identified to genus and species level by MALDI-TOF mass spectrometry (11). In some cases, the identification score is not sufficiently reliable to allow speciation, and next-generation sequencing is required to correctly speciate the isolates. This study describes the characterization of two clinical isolates of *Campylobacter* sent to NRCCH for identification and speciation in 2020 and 2022 by two independent laboratories. Biochemical characterization and mass spectrometry were not able to identify these isolates to species level. However, *16S* rDNA and GyrA phylogeny as well as whole-genome sequence analyses, such as average nucleotide identity (ANI) and DNA-DNA hybridization analyses (DDH), indicated that strains belong to the novel species of *Campylobacter vicugnae* (17–19). Cytopathogenic effects we observed *in vitro* suggest that this species is likely to be a novel human pathogen.

## MATERIALS AND METHODS

### Bacterial strain isolation

Bacteria were grown on Columbia blood agar (CBA) plate with 5% sheep’s blood (Thermo-Fisher Scientific, MA) for 24–48 hours at 25°C, 35°C, or 42°C. Cultures were incubated in sealed jars using an Anoxomat microprocessor (Mart Microbiology, B.V. Lichtenvoorde, The Netherlands), which creates an atmosphere of 80%–90% N_2_, 5%–10% CO_2_, and 5%–10% H_2_. Single colonies for each of the strains were stored at −80°C in brucella broth with 25% glycerol.

### Identification and biochemical characterization

Both strains were assessed by MALDI-TOF mass spectrometry (Bruker, 2023, library with 11,758 spectrum) for identification, as previously described by Bessede et al. (11). Enzymatic activities were assessed by using the API Campy gallery (bioMérieux, Marcy L*'*Etoile, France). The presence of catalase and oxidase was investigated using standard routine methods as previously described (20, 21). Susceptibility to nalidixic acid (30 µg) and cephalothin (30 µg) was assessed by disk diffusion (Biorad, Marnes-La-Coquette, France). Antibiotic susceptibilities to ampicillin, ciprofloxacin, erythromycin, tetracycline, and gentamicin were assessed based on the CA-SFM recommendations (V.2.0. May 2022) (https://www.sfm-microbiologie.org/wp-content/uploads/2022/05/CASFM2022_V1.0.pdf): Mueller-Hinton (MH) agar supplemented with 5% defibrinated horse blood (MH-F) and 20 mg/L β-NAD (bioMérieux, Marcy l’Etoile, France); inoculum: 0.5 McFarland standard; incubation at 35°C for 48 hours in a microaerobic environment as described above. For each strain, inhibition zone diameters were measured (Biorad, Marnes-La-Coquette, France) using the SIRscan Auto (i2A, Montpellier, France), as previously described (22). The reference strain *C. jejuni* ATCC 33560 was used as a control strain, according to CASFM/EUCAST recommendations.

### Microscopy and imaging

The morphology, cell size, and presence of flagella were determined by the transmission electron microscopy. Bacteria were introduced into a fixative solution of 2.5% glutaraldehyde in 0.1 M cacodylate buffer (pH 7.4) and incubated for 1 hours at room temperature. After centrifugation for 3 min at 5,000 rpm, pellets were mixed/suspended in 500 µL of 0.1 M cacodylate buffer (pH 7.4). A volume of 10 µL bacterial suspension was adsorbed on carbon grids with negative ionization (Delta Microscopy, Toulouse, France) and negatively stained with a nano-tungsten solution. Grids were examined with a transmission electron microscope (Talos F200S G2, Thermofisher, Eindhoven, The Netherlands) at 200 kV, equipped with a OneView camera (Gatan, Paris, France).

### Genomes sequencing and assembly

DNA was extracted from bacterial cultures using the MagNA Pure 96 DNA and Viral NA SV Kit, and DNA purification was performed from bacterial lysis on a MagNA Pure 96 System (Roche Applied Science, Manheim, Germany). Quantification and purity checks (260/280 and 260/230 ratios) were determined by spectrophotometry (NanoDrop Technologies, Wilmington, DE, USA) before sequencing. Paired-end next-generation sequencing was performed on DNA samples using NovaSeq 6000 (Henri-Mondor APHP, Créteil, France) for both isolates. Additionally, FastQC v0.11.8 (23) was used to ensure quality. Paired-end sequences were trimmed, and genomes were assembled using Sickle v1.33 (24) and SKESA v2.5.1 (25), respectively.

### Gene content analyses

Each analysis was performed from assembled *fasta* files using a Linux (Bash and Python) homemade pipeline. Multilocus sequence typing (MLST) (26) was performed using Blast 2.12.0+ command line tool (27) and the PubMLST *Campylobacter* scheme (28). Blast 2.12.0+ was also used in order to identify resistance and virulence genes, combined with CARD, NCBI, ResFinder, VFDB, and VirulenceFinder databases as well as an in-house *Campylobacter* resistance genes and mutations database constructed from multiple previously published studies (29–34). Genome annotations were performed using Prokka v1.14.6 (35), and a pangenome was built from every .*gff* files using Roary v3.13.0 (36). The pangenome was used to identify shared genes between all studied genomes. *16S rDNA* and GyrA phylogenetic trees were constructed from 60 nucleotide and protein sequences from 48 unique species or subspecies based on LPSN database (https://lpsn.dsmz.de/search?word=Campylobacter). Muscle v3.8.1551 (37) was used for alignments, and the neighbor-joining method from Mega 11 (38) was used to calculate phylogenetic distances (1,000 replicates bootstrap test). Similar tools were used to build phylogenetic trees from Miller et al. (15, 17) and Wang et al. (16) genomes based on concatenated sequence alignments of complete *cdtA*/*B*/*C* operons and all flagella genes that were previously identified (*n* = 26). Every tree was drawn using iTOL v5 (39). Finally, plasmid presence was estimated using RFPlasmid prediction software v0.0.18 (40).

### Whole-genome ANI and DDH

Pairwise average nucleotide identity analysis using FastANI v1.1 (41) and DNA-DNA hybridization analysis using GGDC online tool (http://ggdc.dsmz.de/ggdc.php) were performed to enable whole-genome comparison of the two studied isolates against a set of 92 *Campylobacter* species genomes, including *C. devanensis*, *C. porcelli*, and *C. vicugnae* genomes from Miller et al. and Wang et al. studies (15–17). Precisely, both analyses were performed using a total of 26 *C*. *lanienae*, 36 *C*. *devanensis* (including type strain NCTC 15074T), 4 *C. porcelli* (including type strain NCTC 15075T), 13 *C*. *vicugnae* [including type strain NCTC 15076T and 3 *C. ovis-vicugnae* (16)], 3 *C. fetus* subsp*. fetus*, 1 *C. fetus* subsp*. venerealis*, 2 *C. fetus* subsp*. testudinum*, 3 C. *hyointestinalis* subsp*. hyointestinalis* or *lawsonii*, and 2 *C. iguaniorum*. A significant probability for ANI being >95% (98% for subspecies) and for DDH being >70% was applied to conclude that the two studied genomes belonged to a specific species. All scores were computed into a heatmap using Seaborn 0.12.2 Python module (42).

### Cell culture

The human epithelial cell line Caco-2 (ATCC HTB-37), derived from a colorectal adenocarcinoma, was grown inMinimum Essential Medium (α-MEM) (Gibco, New Zealand) supplemented with 10% heat-inactivated fetal calf serum (Fisher Scientific, Strasbourg, France) at 37°C in a 5% CO_2_ humidified atmosphere. Cells were seeded on glass coverslips in 24 well plates, at a density of 40,000 cells per well, 24 hours before the addition of bacteria, as previously described by Varon et al. (43).

### Coculture experiment

Strains 2020-M and 2022-B were grown to mid-log phase on CBA plate with 5% sheep’s blood (Thermo Fisher Scientific, MA) and incubated at 35°C in a jar. An Anoxomat microprocessor (Mart Microbiology BV, Lichtenvoorde, The Netherlands) created an microaerobic atmosphere of 80%–90% N_2_, 5%–10% CO_2_, and 5%–10% H_2_. Bacteria were then suspended in brain heart infusion broth and diluted to OD_600nm_ of 1, corresponding to a concentration of 10^9^ CFU/mL. Cells were co-cultivated with 2020-M and 2022-B isolates, as well as *C. jejuni* NCTC 11168 as a positive control for 4 hours, at a multiplicity of infection of 100 bacteria per cell. The cells were washed three times with 1 mL of PBS and cultivated for further 2 hours in α-MEM supplemented with 10% heat-inactivated fetal calf serum and 10 µg/mL of gentamicin to kill all extracellular bacterial cells. The coculture was then maintained for 72 hours, as previously described (43), and cells were fixed with 4% paraformaldehyde in PBS and assessed by immunofluorescence.

### Immunofluorescence and imaging

Immunofluorescence staining was performed as previously described (44). Cells were permeabilized with 0.2% Triton X100 in PBS and blocked using 10% FCS and 1% BSA in PBS to prevent non-specific antibody binding. The cells were then incubated with primary antibody (anti-γH2AX - Cell Signaling, #9718) and Alexa Fluor 488-labeled secondary antibody (Invitrogen, # A-21206) and diluted at 1/100 and 1/400, respectively, in 10% FCS in PBS. Counterstaining of nuclei and actin cytoskeleton was performed using 4*'*,6*'*-diamidino-2-phenylindole (DAPI, blue) and AlexaFluor 647-labeled anti-phalloidin antibody. Epifluorescence imaging was performed using Eclipse 50i epi-fluorescence microscope (Nikon, Champigny sur Marne, France) equipped with the DP23-M camera and the CellSens Standard software (Olympus, Tokyo, Japan), Nikon 20× objective.

## RESULTS

### Clinical data

Strain 2020-M was isolated in 2020 from the feces of a 50-year-old immunocompromised male patient with gastroenteritis, at Saint Antoine Hospital (APHP, Paris, France). Strain 2022-B was isolated by a laboratory in the Deux-Sèvres area in France in 2022 from the feces of a 68-year-old female patient suffering from gastroenteritis with bloody liquid stools, fever, and abdominal pain for 3 weeks. Both patients have neither recollection of recent travel nor contact with animals.

### Biochemical and growth characteristics

For both isolates, bacterial cells were motile, curved, and gram negative, with translucent and shiny colonies, typical of *Campylobacter* spp. MALDI-TOF analysis failed to speciate these isolates which indicated that they may belong to an unusual species absent from the database. There was no visible growth in a CO_2_ enriched or anaerobic atmosphere. Strain 2020-M and 2022-B colonies were visible on trypticase soy agar plates following incubation at 35°C after 24 hours under microaerobic conditions (Table 1). There was no growth at 25°C or 42°C for both strains. Catalase and oxidase activities were detected. API Campy gallery showed that both strains were positive for nitrate reduction and alkaline phosphatase. There was no urease, hippuricase, or γ-glutamyl transpeptidase activity. (Table 1). These profiles are fully similar to *C. lanienae* type strain NCTC 13004. However, the absence of TTC reduction and γ-glutamyl transpeptidase activity for our two isolates is inconsistent with the results published by Miller et al. for *C. vicugnae* (17) and by Wang et al. for *C. ovis-vicugnae* (16), respectively. According to antibiotic susceptibility testing, both isolates were susceptible to all antimicrobials tested *in vitro* (i.e., ampicillin, ciprofloxacin, erythromycin, tetracycline, and gentamicin) as described for *C. ovis-vicugnae* (16).

**TABLE 1:** Phenotypic characteristics that differentiate the two studied isolates from nine *C. vicugnae* isolates (Miller et al.), three *C. ovis-vicugnae* (Wang et al.), one *C. lanianae,* and one *C. hyointestinalis* subsp. *lawsonii*^*a*^

Characteristics	2020-M and 2022-B	*C. vicugnae* (*n = 9*) (Miller et al.)	*C. ovis-vicugnae* (*n = 3*) (Wang et al.)	*C. lanienae* NCTC 13004	*C. hyointestinalis* subsp. *lawsonii*
Growth temperature range (°C)	35	37–42	37	37–42	[18–25]
Atmospheric requirements	mO_2_	mO_2_	mO_2_	mO_2_ (ANO_2_)^*b*^	42 mO_2_
Oxidase	+	+	+	+	+
Catalase	+	+	+	+	+
Nitrate reduction	+	+	+	+	+
Esterase	+	Untested	+	+	+
Urease	−	−	−	−	−
Alkaline phosphatase	+	+	+	+	+
Hippurate hydrolysis	−	−	−	−	−
TTC reduction	−	+	−	−	+
GGT	−	Untested	+	−	−
Resistance to: nalidixic acid (30 mg)	+	+	+	+	+
Resistance to: cephalothin (30 mg)	+	Variable	Untested	+	F
DNA G + C content (mol%)	32.8; 34.5	30–32.07	31.9–33.1	35	35–36

^*a*^ +, positive result; −, negative result; mO_2_, microaerobic condition; ANO_2_, anaerobic condition.

^*b*^ *, weak growth; [], 50%–60 % strains grow at this temperature; F, 7%–27 % strains positive.

### Morphological characteristics

**Fig 1:**
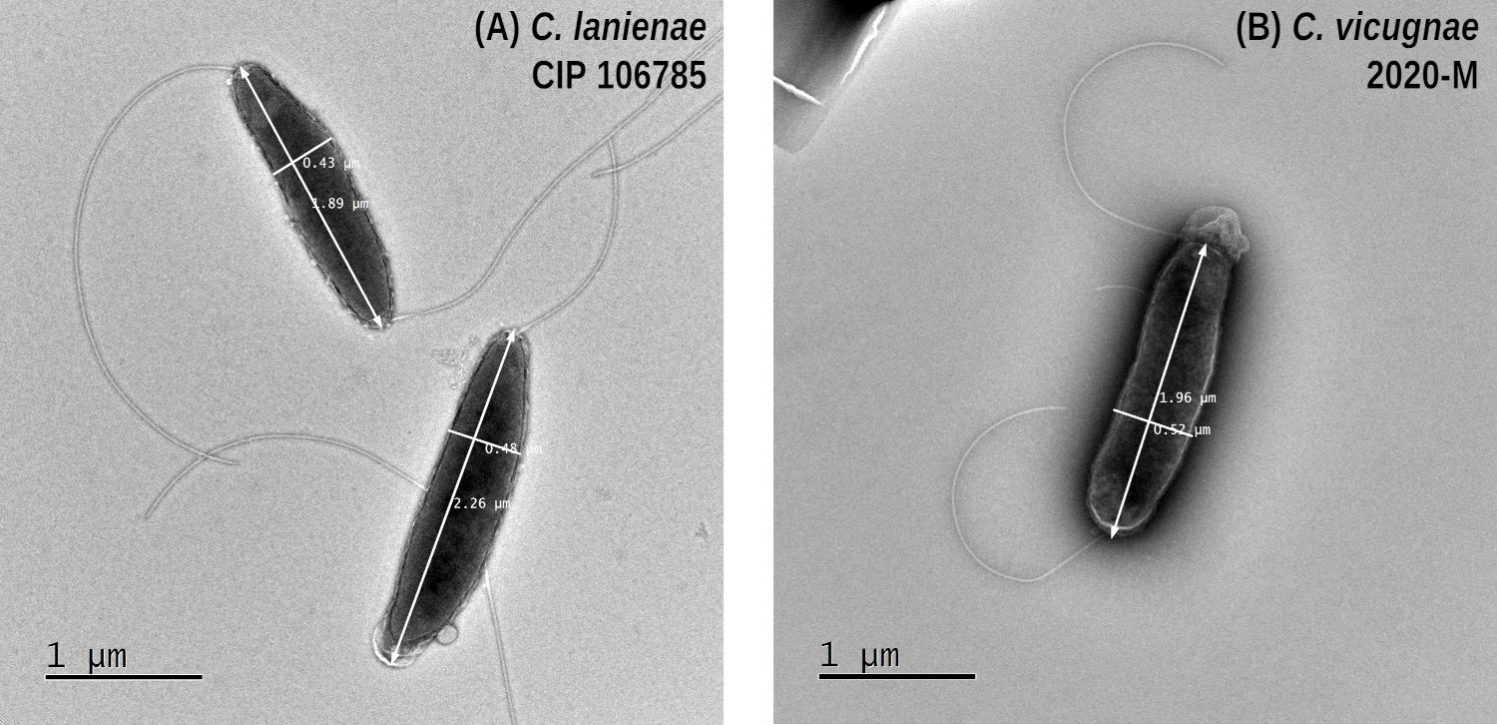
Electron microscopy using the reference strain *C. lanienae* CIP 106785/NCTC 13004 (**A**) and the 2020-M strain (**B**). Both cells have similar morphologies, namely an average size of around 2 × 0.5 µm, amphitrichous, and with unsheathed flagella.

TEM microscopic observation of strain 2020-M revealed a rod-shaped bacterium, approximately 2.2 µm long and 0.45 µm wide (Fig. 1). Two amphitrichous unsheathed flagella were visible. This morphology was similar to that of *C. lanienae* and *C. vicugnae* NCTC 15074T described by Miller et al. (17) and *C. ovis-vicugnae* SYS25-1 described by Wang et al. (16).

### Genome content, phylogeny, and taxonomy

**Fig 2:**
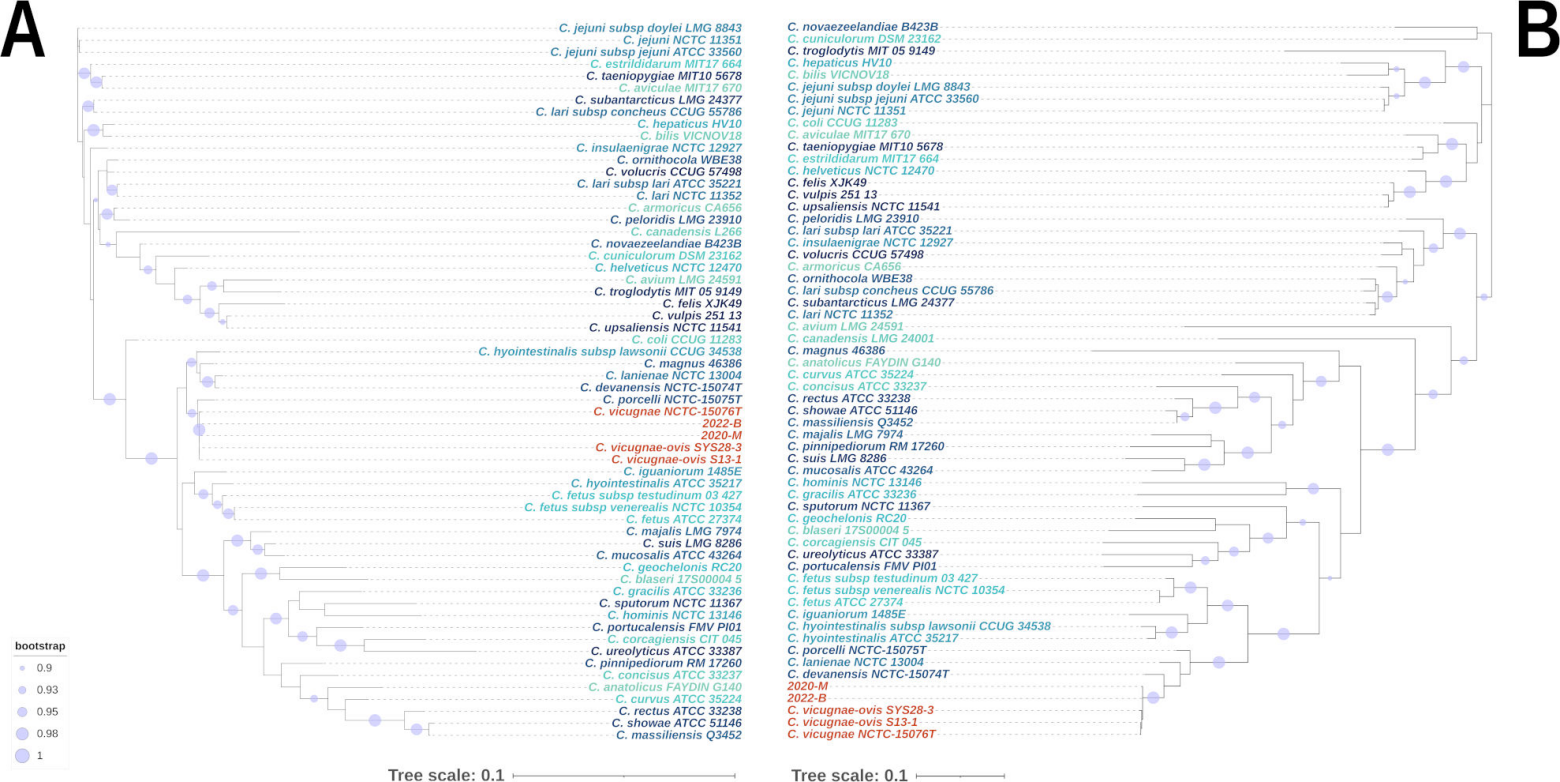
Phylogenetic tree of *16S rDNA* nucleotide (**A**) and GyrA protein (**B**) sequences. Both trees were constructed from various *Campylobacter* species or subspecies. Specifically, *16S rDNA* tree (**A**) and GyrA (**B**) are based on the alignment of 60 sequences from 48 unique species or subspecies. *C. vicugnae* isolates are highlighted in red.

**Fig 3:**
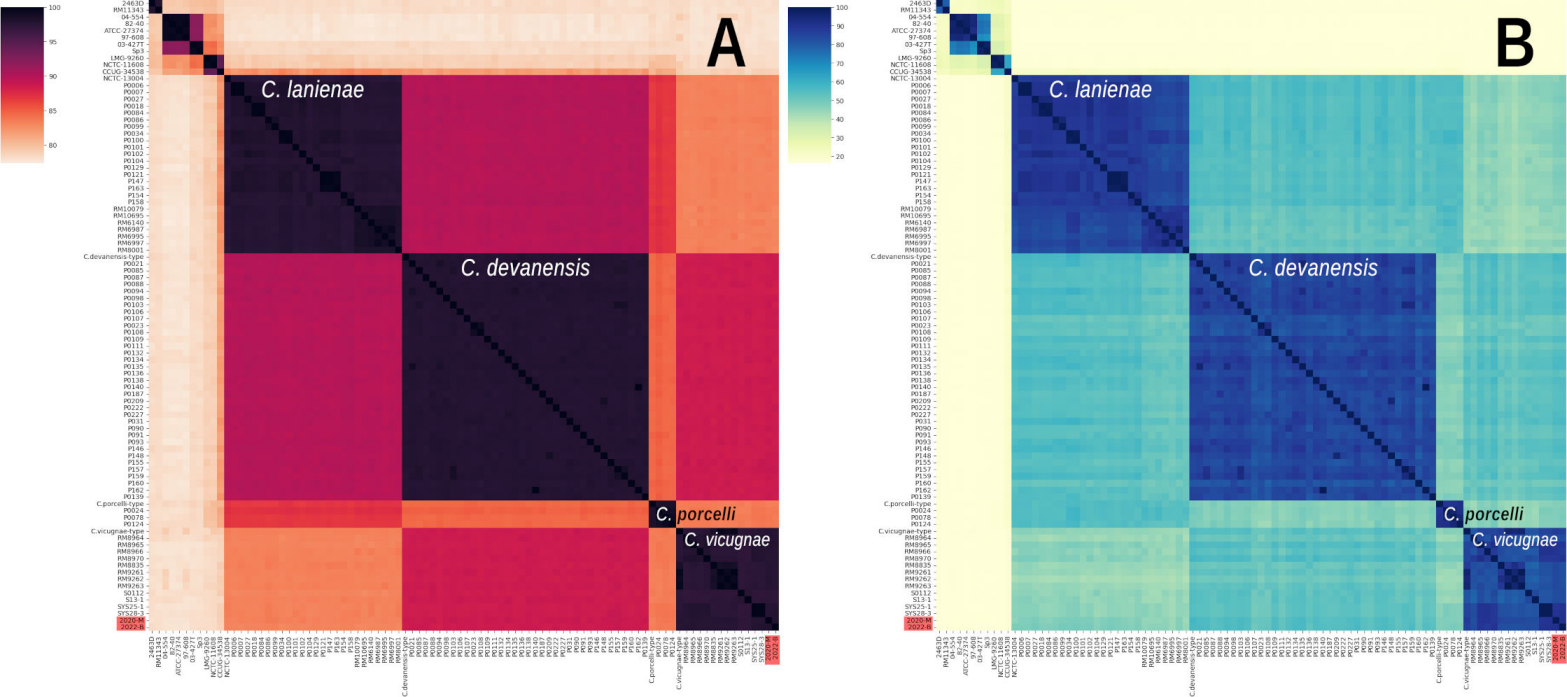
ANI (A) and DDH (B) analyses performed on 2020-M and 2022-B strains. Studied clinical isolates are highlighted in red. The strains described in the studies of Miller et al. and Wang et al. were analyzed, including type strains *Campylobacter devanensis* sp. nov. NCTC 15074T, *Campylobacter porcelli* sp. nov. NCTC 15075T, and *Campylobacter vicugnae* sp. nov. NCTC 15076T, marked with “*type”* in both axes. The highly significant ANI and DDH values indicate that strains from different clusters do not belong to the same *Campylobacter* species, as suggested by Miller et al.

The 2020-M genome consisted of 1,541,227 bp for a GC% of 31.3% and contained 1,535 coding sequences (CDS; Prokka annotation). The 2022-B genome consisted of 1,530,584 bp for a lower GC% of 30.6% and contained 1,521 coding sequences. These data are comparable to *C. vicugnae* genomes from Miller et al. study (17), which consisted, on average (*n* = 10 genomes), of a genome size of 1,615,213 bp, a GC% of 30.9%, and a number of coding sequences of 1,635, as well as to *C. ovis-vicugnae* genomes from Wang et al. study (16) (*n* = 3 genomes): 1,537,463 bp genome size, a GC% of 32.4%, and a number of coding sequences of 1,533. No plasmid was identified in both 2020-M and 2022-B isolates using RFPlasmid. Prokka annotation and pangenome design using Roary revealed that 2020-M and 2022-B genomes shared only 7% orthology based on their CDS with *C. lanienae* type strain NCTC 13004, approximately 73% ± 3% with two isolates from *C. vicugnae* (type strain NCTC 15076T and RM8964) and 76% ± 4% with three *C. ovis-vicugnae* genomes (Table 2). *16S* rDNA as well as GyrA-based phylogenetic analyses allowed clustering of the two studied strains with *C. vicugnae* and *C. ovis-vicugnae* isolates, with bootstrap scores over 95% and close to *C. porcelli* NCTC 15075T, *C. devanensis* NCTC 15074T, *C. lanienae* NCTC 13004, *C. magnus* 46386, and *C. hyointestinalis* subsp. *lawsonii* (Fig. 2). ANI and DDH analyses are shown as heatmaps in Fig. 3 (complete pairwise score matrices are provided in Table S1). ANI analyses revealed that both studied isolates had validated ANI percentages of 98%, on average, against *C. vicugnae* genomes from Miller et al. (17) and Wang et al. (16) (Table 2). In contrast, both isolates shared an ANI value of 97.95% similarity with each other. DDH analyses confirmed the results of ANI, with 95% similarity. For strains 2020-M and 2022-B, all DDH scores against *C. vicugnae* were considerably higher than the speciation threshold of 70%, with 87% in average. ANI and DDH heatmaps showed that *C. vicugnae*, *C. porcelli,* and *C. devanensis* genomes had non-valid ANI and DDH values against each others, supporting Miller et al. study, suggesting that these three clades of isolates are indeed three novel species of *Campylobacter* close to *C. lanienae* (17).

**TABLE 2:** Comparisons between the genomes of 2020-M and 2022-B and the reference genome of C. lanienae, two strains of C. vicugnae (type strain NCTC 15076T and RM8964 from Miller et al.), and three C. ovis-vicugnae isolates (S13-1, SYS25-1, and SYS28-3 from Wang et al.)^*a*^

A	2020 M	2022-B	C. lanienae NCTC 13004	C. vicugnae NCTC 15,076T	C. vicugnae RM8964	C. ovis-vicugnae S13-1	C. ovis-vicugnae SYS25-1	C. ovis-vicugnae SYS28-3
Genome size (bp)	1,541,227	1,530,584	1,594,554	1,612,610	1,729,336	1,442,012	1,580,362	1,590,016
GC %	31.3	30.6	35	30	32	32.1	33.1	31.9
CDS numbers^*b*^	1,535	1,521	1,596	1,648	1,743	1,455	1,568	1,576
CRISPR•Cas operon n	2	2	1	1	2	1	1	1
Plasmids n (size in kb.)	*n* = 0	*n* = 0	*n* = 0	*n* = 2 (25; 4)	*n* = 1 (25)	*n* = 0	*n* = 0	*n* = 0

B	2020 M	2022-B	C. lanienae NCTC 13004	C. vicugnae NCTC 15,076T	C. vicugnae RM8964	C. ovis-vicugnae S13-1	C. ovis-vicugnae SYS25-1	C. ovis-vicugnae SYS28-3
2020 M		80 **• 98 • 95**^*c*^	6.8 • 84 • 60	74 **• 98 • 91**	69 **• 98 • 82**	70 **• 97 • 83**	77 **• 98 • 89**	76 **• 98 • 89**
2022-B	80 **• 98 • 95**		7 • 83 • 67	76 **• 98 • 90**	72 **• 98 • 85**	74 **• 97 • 83**	80 **• 98 • 90**	81 **• 98 • 90**
C. lanienae NCTC 13004	6.8 • 84 • 60	7 • 83 • 67		7 • 84 • 63	7 • 83 • 58	9 • 83 • 46	9 • 83 • 46	9 • 83 • 46
C. vicugnae NCTC 15,076T	74 **• 98 • 91**	76 **• 98 • 90**	7 • 84 • 63		68 **• 98 • 81**	66 **• 97 • 82**	72 **• 97 • 86**	72 **• 97 • 86**
C. vicugnae RM8964	69 • **98 • 82**	72 **• 98 • 85**	7 • 83 • 58	68 **• 98 • 81**		66 **• 97 • 77**	72 **• 98 • 83**	71 **• 98 • 83**
C. ovis-vicugnae S13-1	70 **• 97 • 83**	74 **• 97 • 83**	9 • 83 • 46	66 **• 97 • 82**	66 **• 97 • 77**		74 **• 97 • 82**	73 • **97** • 81
C. ovis-vicugnae SYS25-1	77 **• 98 • 89**	80 **• 98 • 90**	9 • 83 • 46	72 **• 97 • 86**	72 **• 98 • 83**	74 **• 97 • 82**		98 **• 100 • 100**
C. ovis-vicugnae SYS28-3	76 **• 98 • 89**	81 **• 98 • 90**	9 • 83 • 46	72 **• 97 • 86**	71 **• 98 • 83**	73 • **97** • 81	98 **• 100 • 100**	

^*a*^ Global data on genome content.

^*b*^ Pairwise **CDS** (% of similar genes).

^*c*^ **ANI** • **DDH** comparisons of all eight genomes: significant ANI and DDH values are marked as bold (significance level for ANI ≥ 95%; significance level for DDH ≥ 70%).

### Antibiotic resistance and virulence gene evaluation

**Fig 4:**
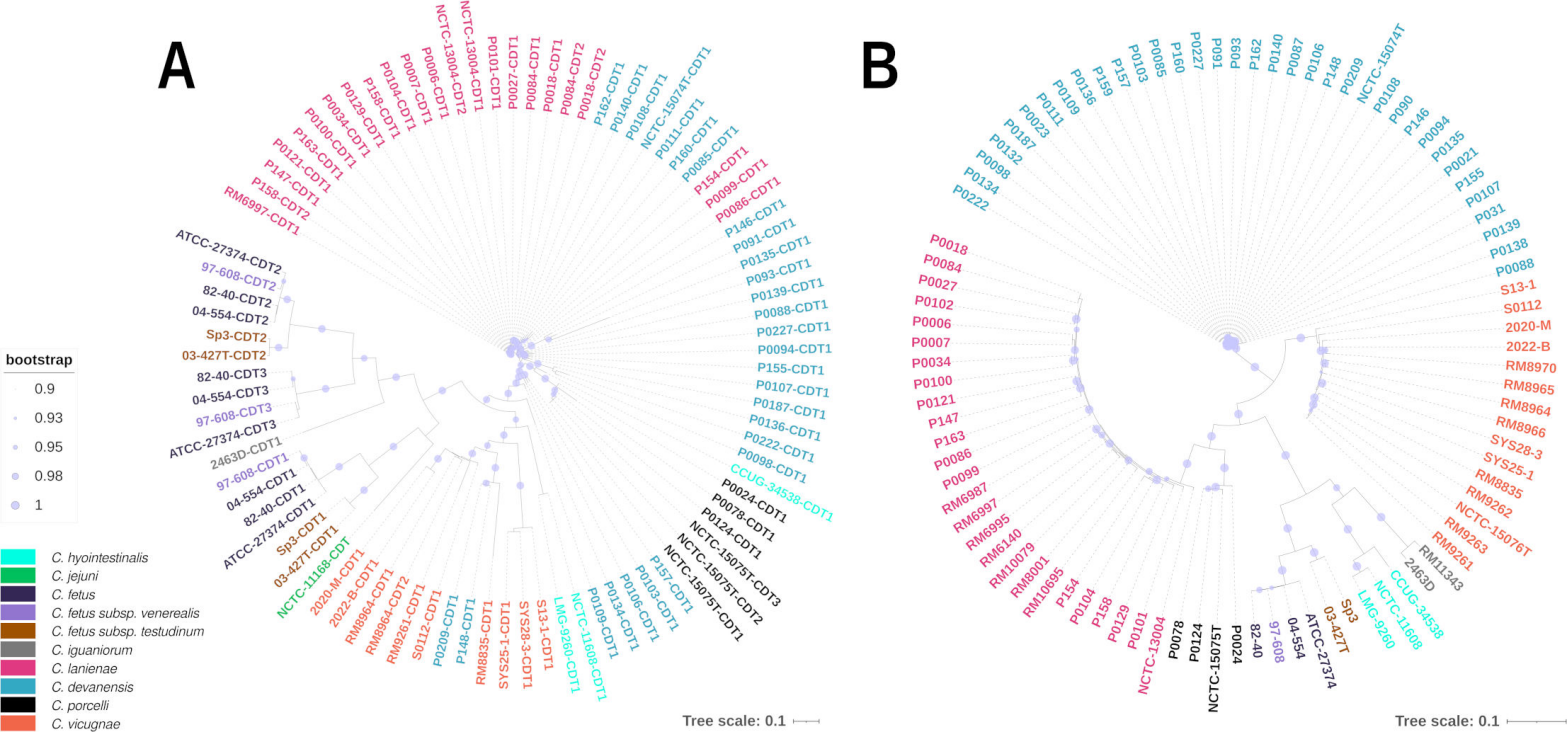
Phylogenetic tree constructed from concatenated sequence alignments of *cdtA*, *cdtB*, and *cdtC* (**A**) and concatenated flagella genes (**B**). (**A**) Several isolates were displaying more than one *cdtA*/*B*/*C* operon (up to three). Only complete copies of the *cdtA*/*B*/*C* operon in the present tree are shown, and incomplete *cdt* operons were not taken into account. *C. vicugnae* in orange here possess only one copy of *cdtA*/*B*/*C*. Similar to the ANI and DDH analyses, the two studied human isolates cluster together among *C. vicugnae* isolates. *C. lanienae* and *C. devanensis* isolates showed closely related CDT operons, while *C. porcelli* and *C. vicugnae* displayed more diverse sequences. (**B**) A total of 26 flagella genes were found among all strains (*fliM, fliI, fliG, fliN, flgC, flgG, fliP, flhA, fliQ, flhG, flgR, flgP, fliA, fliS, flhB, flgB, flgG2, fliR, flgJ, flhF, flgH, fliW, flgI, flgS, motA, ptmB*). Phylogeny based on concatenated flagella genes revealed clear segregation between all species.

**Fig 5:**
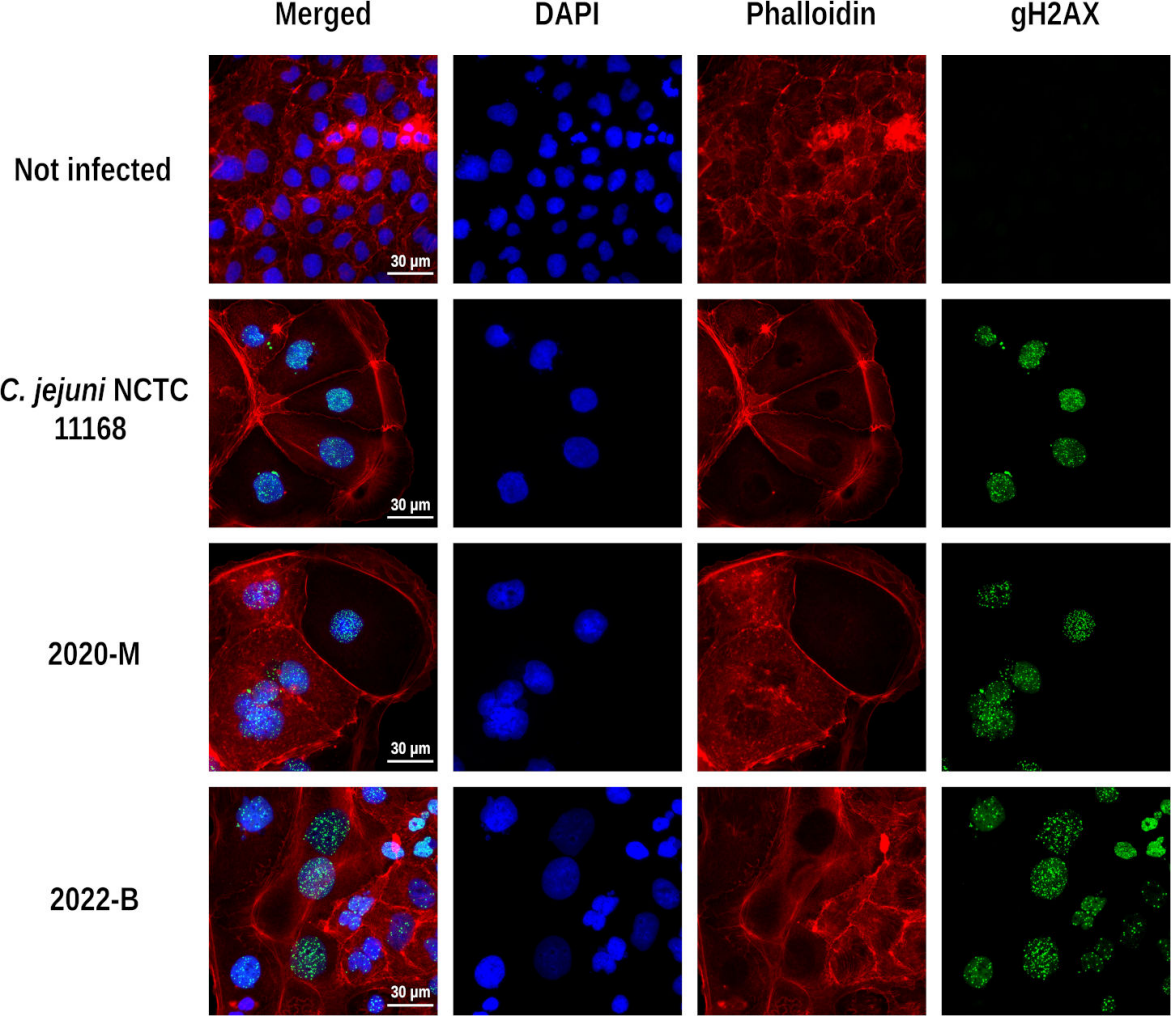
*In vitro* effects of *C. jejuni* NCTC 11168, 2020-M, and 2022-B isolates on human intestinal epithelial cells. Cells were stained with fluorescent primary antibody and secondary antibody targeting phosphorylated (phospho S139) γH2AX (green), and DAPI (blue) and phalloidin (red) to counterstain the nucleus and actin cytoskeleton, respectively. The top row shows uninfected Caco-2 cells control. The DAPI and phalloidin staining show enlarged nuclei and cytoplasm, respectively, in the three infected conditions as well as a positive staining for DNA damage (γH2AX, green foci).

No molecular resistance mechanisms were identified via public antibiotic resistance gene databases within both studied isolates, suggesting susceptibility to commonly used antimicrobial molecules. In contrast, *C. lanienae* type strain NCTC 13004 harbors tetracycline resistance gene *tet*(O). Notably, Prokka annotation showed that both isolates described in this study contained one copy of a complete cytolethal distending toxin (CDT) operon, comprising *cdtA*, *cdtB,* and *cdtC*, with nucleotide sequences similar to other *C. vicugnae* CDT operons (Fig. 4A, CDT peptide sequences are available in Table S2). Co-culture of 2020-M and 2022-B strains with Caco-2 human cell line led to DNA damage, giving rise to cellular and nuclear enlargement associated with a profound cytoskeleton remodeling (Fig. 5), as observed for *C. jejuni* NCTC 11168 control strain. These effects are typical of exposure to CDT, often encoded by campylobacters and other gram-negative bacteria, indicating that strains 2020-M and 2022-B both produce active CDT when in contact with human cells. It was interesting to note that the cytotoxic effects on the Caco-2 cells could be observed within 48 hours (data not shown) and were profound by 72 hours post-infection, as compared to non-infected control.

Overall, the molecular identification from vfdb and VirulenceFinder databases highlighted 66 virulence genes among the 90 related *Campylobacter* spp. genomes (15) and the strains described in this study (Table 3, full genes list and their distributions among each isolate are shown in Table S2). A total of 26 genes were related to flagella, 16 to capsule, colonization, and invasion capacity, 10 to cell surface and immunity, 6 to efflux system, 4 to chemotaxis, and 4 to growth and regulation. For each category, *C. vicugnae* genomes (*n* = 15) displayed on average 21, 9, 1, 3, 4, and 3 genes, respectively. Flagella genes were highly prevalent among all studied genomes (with an average of 20 genes per strains from *fliM*, *fliI*, *fliG*, *fliN*, *flgC*, *flgG*, *fliP*, *flhA*, *fliQ*, *flhG*, *flgR*, *flgP*, *fliA*, *fliS*, *flhB*, *flgB*, *flgG2*, *fliR*, *flgJ*, *flhF*, *flgH*, *fliW*, *flgI*, *flgS*, *motA* and *ptmB*), and concatenated nucleotide sequences alignment allowed to clearly segregate each studied species or subspecies as shown in Fig. 4B.

**TABLE 3:** Average virulence genes for each species or subspecies^*a*^

		Average *n* of virulence genes (db = VirulenceFinder and vfdb) classified by gene function							
Group	No. of strains	CDT operons	Flagella (*n* = 26)	Capsule, colonization, invasion (*n* = 16)	Cell surface, immunity (*n* = 10)	Efflux system (*n* = 6)	Chemotaxis (*n* = 4)	Growth, regulation (*n* = 4)	Total average
*C. fetus* or subspecies	6	2.7	20.5	4.67	1.17	1.17	4	2	33.5
*C. hyointestinalis* or subspecies	3	1	20.67	3.33	0.67	1	4	1.67	31.33
*C. iguaniorum*	2	0.5	23.5	1.5	1	2.5	4	1	33.5
*C. lanienae*	26	1	18.77	7.81	1.5	3.65	4	1.12	36.85
*C. devanensis*	36	0.8	16.03	8.36	1.17	2.36	4	1.36	33.28
*C. porcelli*	4	1.5	16.5	9.25	1	3	4	0.5	34.25
*C. vicugnae* (Miller et al*.*)	10	0.8	20.7	9.1	1.2	3	4	2.4	40.4
*C. ovis-vicugnae* (Wang et al*.*)	3	1	20.67	9.33	1.33	2.33	4	3	40.67
2020-M	1	1	21	8	1	2	4	3	39
2022-B	1	1	20	8	1	3	4	3	39

^*a*^ Virulence genes identified using Blast 2.12.0+ command line tool combined with VirulenceFinder and vfdb databases shown here are arbitrarily gathered into various functions groups: flagella (*fliM, fliI, fliG, fliN, flgC, flgG, fliP, flhA, fliQ, flhG, flgR, flgP, fliA, fliS, flhB, flgB, flgG2, fliR, flgJ, flhF, flgH, fliW, flgI, flgS, motA, ptmB*), capsule, colonization, and invasion (*kpsT, gmhA2, rfbC, gmhA, kpsM, kpsE, kpsD, Cj1427c, hldD, Cj1419c, gmhB, Cj1416c, Cj1420c, ciaB, Cj1135, hldE*), cell surface and immunity (*neuB1, glf, hddC, hddA, pebA, neuC1, Cj1279c, Cj1433c, Cj1440c, Cj1138*), efflux system (*pseB, pseI, pseC, pseF, pseA, pseH*), chemotaxis (*cheA*, *cheV*, *cheY*, *cheW*), and growth and regulation (*fcl, Cj0883c, rpoN, cysC*). Full distribution of each genes by isolates along with Genbank accession numbers is available in Table S2.

## DISCUSSION

In the present study, we describe the characterization of two *Campylobacter* strains isolated 2 years apart from the stools of French patients with intestinal disorders. We demonstrate that these two strains are similar to a recent novel species, described as “*Campylobacter vicugnae”* by Miller et al*.* (15, 17) and as “*Campylobacter ovis”* by Wang et al. (16). The genomes of the two strains described in the present study are similar to that of Miller et al*.* and Wang et al. studies. *C. vicugnae* is distinguished by a reduced genome size ranging from 1.44 to 1.73 Mb (*n* = 15 genomes included in the present study) compared to other *Campylobacter* species. GC% content is also in agreement, ranging from 30% to 33.1% GC. *C. vicugnae* isolates may also harbor distinct CRISPR/Cas systems and carry 20–21 unlinked and similar, but non-identical, flagellin genes scattered throughout the chromosome, which is also the case for the two strains described in this study. In contrast to Miller et al*.* and Wang et al. study, and based on enzymatic activities assessed using the API Campy gallery, *C. vicugnae* strains do not display any TCC reduction and γ-glutamyl transpeptidase activity, respectively. Moreover, no gene related to any γ-glutamyl transpeptidase activity was found among 2020-M, 2022-B, or within genomic data reported by Miller et al*.* and Wang et al.

As shown here, WGS analysis was employed to accurately speciate strains 2020-M and 2022-B using genome-to-genome comparisons, such as ANI and DDH. Based on the positive ANI and DDH results between the two 2020-M and 2022-B strains, as well as their identical phenotypic profiles, it can be assumed that, at the very least, the two strains are indeed similar. Furthermore, ANI analyses assigned the two study strains to *C. vicugnae* with scores above the speciation threshold of 95%. Miller et al. initially suggested that the ANI species boundary in *Campylobacter* is lower than 95% and that ANI values between clade 1, 2, or 3 and the other taxa within the *C. fetus* group are between 71% and 82% (15), which they recently confirmed (17). Based on these data and those of Wang et al. study, we agree with the conclusion which suggests that *C. vicugnae* is a valid novel species.

Sequence comparison of the A, B, and C genes of the CDT operon from the NCBI database showed that few other genomes of *Campylobacter* strains isolated in 2009 in the USA possessed similar sequences to that encoded by 2020-M and 2022-B strains (15). These sequences were not initially displayed in Wang et al*.* study. CDT phylogeny revealed a relationship between nucleotide sequences of the two strains in this study and other *C. vicugnae* isolates (Fig. 4). CDT is a bacterial toxin found in many gram-negative pathogenic bacteria (45). CDT comprises three subunits: CdtA, CdtB, and CdtC. CdtA and CdtC bind to the cell membrane-associated lipid raft (46), and CdtB is then transported into the host cells, which induce DNA breakage followed by the cell cycle arrest at G2/M (47). The presence of a complete and intact CDT operon in the genome of the two clinical strains described in the present study, encoding for a functional CDT toxin, argues in favor of the virulence of *C. vicugnae*. As shown for other related species, such as *H. pullorum*, the CdtB plays a major role in its cytopathogenic effects (43), which suggests that it is a crucial virulence factor involved *in vivo* targeting the integrity of the intestinal barrier. Nevertheless, we could not identify the presence of a unique, complete, and preserved CDT operon within the genome of each *C. vicugnae* isolate, as displayed in Table S2. According to our study and Miller et al*.* (17), multiple but also degenerated CDT loci could be identified in some *C. vicugnae* isolates. A complementary study may be required in order to investigate the different level of CDT activities among every isolates. Although virulence-associated genes are yet to be assigned for *C. vicugnae*, the presence of more than 20 potential virulence genes within the genomes of both described isolates is in agreement with the fact that *C. vicugnae* strains display the larger virulence arsenal as compared to the closest species, such as *C. porcelli*, *C. devanensis,* and *C. lanienae* (Table 3). This study clearly demonstrates that the two *C. vicugnae* strains 2020-M and 2022-B were associated with gastroenteritis in humans and exhibited well-known pathogenic CDT effects, showing that this species can be virulent in humans. This constitutes a first report of isolation of *C. vicugnae* in humans.

The source of infection for the two human cases described in the present study remains unknown. According to the publication of Wang et al*.* (16), isolates of *C. vicugnae* have been sampled from sheep, whereas isolates from Miller et al. study were isolated from goats and alpacas in California, USA (15). In contrast, isolates of *C. porcelli* and *C. devanensis* from this study were mainly sampled from pigs in Scotland. Direct contact with these sources or the consumption of contaminated food, such as sheep meat or milk, could therefore be possible. Interestingly, the Deux-Sèvres district, where the patient infected with the 2022-B strain lives, has France’s leading goat industry in terms of number of goats and volume of milk produced. Unfortunately, the epidemiological questionnaires performed by the two clinical laboratories that isolated these two strains from human samples failed to obtain more precise information.

This work reflects the difficulties in identifying new species of *Campylobacter* by using phenotypic methods only. Campylobacters are able to colonize many sites of the digestive tract (saliva, stomach, cecum, colon, and liver) in various hosts (mammals, birds, and reptiles) leading to constant adaptation of Campylobacteracae to novel niches over time. These adaptation capacities are reflected in gene acquisition and divergent gene evolution, and constitute the main obstacle in determining *Campylobacter* spp. classification, making genome sequencing and bioinformatics valuable tools for Campylobacteracae classification. Isolation of these two *C. vicugnae* strains from human patients highlights the fact that they are likely to be pathogenic to humans exposed to, so far, undetermined contaminated sources and may represent a significant public health threat.

## Data Availability

Raw sequencing data for isolates 2020-M and 2022-B are available under ENA accession numbers ERR13133275 and ERR13133276, respectively. Strains were deposited to Pasteur Institut Collection and to Culture Collection University of Göteborg under the following numbers: CIP 111447 + CCUG 77442 for strain 2020-M and CIP 112448 + CCUG 77443 for strain 2022-B.

## SUPPLEMENTAL MATERIAL

Table S1 spectrum.01523-24-s0001.xlsx ANI and DDH full result table.

Table S2 spectrum.01523-24-s0002.xlsx Full virulence result table.

ASM does not own the copyrights to Supplemental Material that may be linked to, or accessed through, an article. The authors have granted ASM a non-exclusive, world-wide license to publish the Supplemental Material files. Please contact the corresponding author directly for reuse.
